# Criteria for referral to a hospital palliative care team in a cancer setting: a Delphi study of oncologists

**DOI:** 10.1186/s12904-026-02081-5

**Published:** 2026-04-01

**Authors:** Gaurav Chanana, Megha Pruthi, Naveen Salins, Ramandeep Arora

**Affiliations:** 1 Department of Pain and Palliative Medicine, Max Health Care, Delhi, India; 2 Department of Pain and Palliative Medicine, Fortis Memorial Research Institute, Sector 44, Gurugram, Haryana 122002 India; 3 Department of Palliative Medicine and Supportive Care, Kasturba Medical College, Manipal Academy of Higher Education, Manipal, Karnataka 576104 India; 4 Department of Paediatric Oncology, Max Health Care, Delhi, India

**Keywords:** Cancer, Oncologists, Referral, Hospital Palliative Care team, Delphi method

## Abstract

**Background:**

Providing palliative care alongside cancer treatments enhances the quality of life and satisfaction with care for patients with cancer and their families. Determining the timing of referral can often be challenging, as oncologists typically make these decisions. We conducted a Delphi study to establish criteria for referring patients to hospital palliative care teams (HPCT).

**Methods:**

Eighty-five oncologists were presented with 22 criteria developed from literature synthesis, categorised into seven domains, for referral to HPCT. Consensus development was predetermined, with a score of ≥ 7, as agreed upon by over 80% of the participants.

**Results:**

In the first round, 63 out of 85 participants (74%) responded, and in the second round, 41 out of 63 (65%) responded. During the initial round, consensus was reached on 14 out of 22 statements. Six statements were dropped, and two were revised for the next round. The final 14 criteria for HPCT referral were consolidated into ten criteria across five domains: (1) Cancer-related (stage 4 cancer, life expectancy of less than six months), (2) Treatment-related (failed two or more lines of treatment), (3) Symptom-related (ECOG ≥ 3, multiple symptoms including pain and comorbidities affecting patient quality of life, with scores of four or higher on the distress thermometer), (4) Patient preferences (requested palliative care, refused cancer-directed treatment, requested limitation of life-sustaining treatment), and (5) End-of-life care (perceived need for end-of-life care planning).

**Conclusion:**

Developing a palliative care referral criterion provides a structured foundation for identifying cancer patients who might benefit from HPCT referral. Additional empirical validation is required to improve its clinical relevance and outcomes.

## Introduction

The World Health Organisation (WHO) has recognised palliative care as an essential health service and a crucial component of Universal Health Coverage (UHC) [1]. As chronic diseases continue to burden low- and middle-income countries (LMICs) like India, palliative care is becoming increasingly significant in their healthcare systems [2]. The Lancet Commission report reveals that 80% of global serious health-related suffering (SHS) occurs in LMICs, with India having 7.2 million patients experiencing unmet suffering [3]. According to the Worldwide Palliative Care Alliance 2020, India is categorised as “with isolated palliative care provision” at level 3a [4]. The need for improving access to palliative care for patients with cancer is critical, as cancer is expected to affect 29.8 million individuals in India by 2025 and is one of the leading contributors to SHS [5].

Integrating palliative care with oncology is beneficial for patients and caregivers, resulting in improved health outcomes and a higher quality of life [6, 7]. Both the American Society of Clinical Oncology (ASCO) and the European Society of Medical Oncology (ESMO) recommend early consultation with a specialist palliative care team, especially for patients with advanced cancer, ideally within eight weeks of diagnosis [8, 9]. However, despite the availability of palliative care services in many institutions, not all patients who could benefit from it are referred promptly [10, 11]. A recent systematic review identified several barriers to engagement with palliative care, including the absence of dedicated teams, stigma surrounding referrals, presuppositions by oncologists, unclear referral criteria, and concerns about mixed messages [12]. To address these challenges, referral criteria for specialist palliative care have been established by organisations such as the National Comprehensive Cancer Network (NCCN) and ESMO, which have been shown to increase referrals, reduce hospital readmission rates, and decrease ICU use [13, 14]. Although evidence-based referral criteria for palliative care exist, referrals remain infrequent and often occur late in the illness trajectory [15, 16]. It is attributed to factors such as clinician uncertainty, prognostic ambiguity, and the perception that palliative care is only appropriate near the end of life, rather than as a concurrent, supportive service [15, 16].

Due to varying socio-cultural factors and a diverse healthcare system in India, it was essential to establish clear guidelines for referring patients with cancer to palliative care that are relevant to our context. To meet this need, we conducted a modified Delphi study involving oncologists. We aimed to develop socio-culturally appropriate referral criteria, codesigned by the oncology and hospital palliative care teams (HSPT), and agreed upon by the oncologists. These criteria may help oncologists identify patients who need palliative care based on objective triggers, thereby facilitating the development of suitable care pathways and standard operating procedures for policymakers, administrators, and clinicians.

## Methods

### Setting

Nine private hospitals in North India with cancer services were recruited as study centres.

### Formulating the first set of criteria

The core team consisted of two palliative care specialists working in a cancer setting and an oncologist with expertise in Delphi study design. A review of published literature was conducted to identify relevant studies related to the criteria for referral to palliative care and guidelines from leading oncology and palliative care societies, such as the ESMO, ASCO, and NCCN. Synthesis of these data sources resulted in a comprehensive list of over 40 referral criteria. After carefully considering the setting, relevance, and feasibility, the list was revised to 22 criteria, each framed as a statement. Feedback was obtained from two independent oncologists and a palliative care physician, and further modifications were made based on their suggestions, resulting in a final list ready for the Delphi study (Table 1). The twenty-two statements were categorised into seven domains: stage of the disease, cancer-directed treatment options, life expectancy, clinical status, hospital episodes, communication, and miscellaneous.

**Table 1:** Initial criteria developed from literature synthesis for the first round of modified Delphi on Hospital Palliative Care Team Referral in a Cancer Setting and Results of rounds one and two of the modified Delphi study for Hospital Palliative Care Team Referral in a Cancer Setting

Statement		Round 1		Revised Statement	Round 2	
		Percentage of score ≥ 7 on the Likert scale	Outcome		Percentage of score ≥ 7 on the Likert scale	Outcome
1.	In a patient with cancer, which is stage 3 and above, the involvement of a palliative care team is recommended.	51%	Statement dropped			
2.	In a patient with cancer, which is stage 4, the involvement of a palliative care team is recommended.	92%	Agreed			
3.	In a patient with brain metastasis or leptomeningeal disease, the involvement of a palliative care team is recommended.	94%	Agreed			
4.	In a patient with cancer who has failed first-line treatment, the involvement of a palliative care team is recommended.	63%	Statement dropped			
5.	In a patient with cancer who has failed two or more lines of treatment, the involvement of a palliative care team is recommended.	94%	Agreed			
6.	In a patient with cancer with multiple symptoms and co-morbidities which are affecting further cancer-directed treatment, the involvement of a palliative care team is recommended.	92%	Agreed			
7.	In a patient with cancer, if the patient/family refuses further cancer-directed treatment, the involvement of a palliative care team is recommended.	94%	Agreed			
8.	“Would you be surprised if this patient with cancer were to die in the next six months?” - If the answer to this question is NO, then involvement of the palliative care team is recommended.	95%	Agreed			
9.	“Would you be surprised if this patient with cancer were to die in the next twelve months?” - If the answer to this question is NO, then involvement of the palliative care team is recommended.	71%	Statement dropped			
10.	In a patient with cancer who has a performance status ECOG ≥ 3, the involvement of a palliative care team is recommended.	84%	Agreed			
11.	In a patient with cancer, when pain is not responding to regular medications and severely affecting the patient’s QOL, the involvement of the palliative care team is recommended.	95%	Agreed			
12.	In a patient with cancer who has a high symptom burden (described as ≥ 3 symptoms including but not limited to pain, breathlessness, nausea, constipation, tiredness, cognitive impairment, depression, anxiety, sleeplessness, drowsiness, or poor appetite) in moderate to severe intensity (≥ 4/10), involvement of palliative care team is recommended.	94%	Agreed			
13.	in a patient with cancer with multiple symptoms and co-morbidities which are affecting the patient’s QOL, the involvement of a palliative care team is recommended.	90%	Agreed			
14.	In a patient with cancer with hospitalisation of > 7 days for cancer-related complications or symptoms, the involvement of a palliative care team is recommended.	53%	Statement Dropped			
15.	In a patient with cancer with hospitalisation of > 14 days for cancer-related complications or symptoms, the involvement of a palliative care team is recommended.	69%	Statement Dropped			
16.	In a patient with cancer who has had two or more ICU admissions in the last one month for cancer-related complications or symptoms, the involvement of a palliative care team is recommended.	74%	The core team decided to revise the statement based on Delphi participant comments	In a patient with cancer, who has had two or more ICU admissions in the last month for cancer-related complications or symptoms (unrelated to treatment toxicity), involvement of palliative care team is recommended.	68%	Statement Dropped
17.	In a patient with cancer who has had two or more unplanned admissions/emergency visits in the last month for cancer-related complications or symptoms, the involvement of a palliative care team is recommended.	71%	The core team decided to revise the statement based on Delphi participant comments	In a patient with cancer who has had two or more unplanned admissions/emergency visits in the last month for cancer-related complications or symptoms (unrelated to treatment toxicity), involvement of a palliative care team is recommended.”	63%	Statement Dropped
18.	In a patient with cancer, where the family/caregivers are not revealing the diagnosis and prognosis to the patient with the likely intention of “protecting” the patient, the involvement of the palliative care team is recommended.	55%	Statement Dropped			
19.	In a patient with cancer who needs end-of-life care planning (discussing advance care plans, establishing goals of care, enhancing illness understanding and prognostic awareness), the involvement of a palliative care team is recommended	94%	Agreed			
20.	In a patient with cancer, if the patient/family requests palliative care, the involvement of a palliative care team is recommended.	98%	Agreed			
21.	In a patient with cancer, if the patient/ caregiver has been experiencing distress (≥ 4 score on the distress thermometer) in the past week, involvement of the palliative care team is recommended.	87%	Agreed			
22.	In a patient with cancer, if the patient/ family requests limitation of treatment or hastened death, involvement of the palliative care team is recommended.	94%	Agreed			

### Delphi process

This study involved two rounds of a modified Delphi survey conducted among oncologists with expertise in medical, surgical, radiation, and paediatric oncology, as well as haemato-oncologists. These professionals held the independent rights to admit and manage patients under their supervision and practice within their specified setting.

During the first round, participants were asked to rate their level of agreement with each statement on a 9-point Likert scale, where “1” indicated “strongly disagree” and “9” indicated “strongly agree.” The level of consensus was set at a score of 7 or higher by over 80% of participants, with the option to suggest additional criteria. Participants were also encouraged to give feedback on the language and content of the statements. The survey stayed open for four weeks, and the core team sent weekly email reminders to those who had not responded.

After the initial round, any statements that did not reach the required level of consensus were either revised or removed entirely. These revised statements were then presented to the same participants for a second round, where they again rated their agreement on a 1–9 Likert scale. Consensus was defined as a score of 7 or higher by at least 80% of the participants, as agreed upon beforehand. Any statements that did not meet this criterion after the second round were removed, and no further rounds were carried out.

### Ethics

The Institute Ethics Committee (IEC) of Max Health Care, Max Super-speciality Hospital, Saket, New Delhi (IEC Registration Number: Department of Health Research, Max Super-speciality Hospital, New Delhi SEC/NEWINST/2023/DL/020) granted a waiver of consent (Approval Number: BHR/RS/MSSH/DDF/SKT-2/IEC/ONCO/24 − 11) as oncologists participated in this study as stakeholders for developing the HPCT referral criteria. Informed consent for participation was not obtained, as the IEC issued a waiver of consent in accordance with item 5.7 of the 2017 National Ethical Guidelines for Biomedical and Health Research involving Human Participants of the Indian Council of Medical Research.

## Results

Eighty-five potential participants (oncologists) were identified across nine hospitals, and an online survey questionnaire containing 22 statements was sent to them for the first round of participation.

In the first round, 63 of 85 participants responded, yielding a response rate of 74%. A significant 70% of the participants had over ten years of experience in their field. Most participants (43%) specialised in surgical oncology, followed by medical and radiation oncology (22% each), with others from various cancer specialities, including paediatric oncology, haematology-oncology, and gynaecology-oncology. After round one, 14 out of 22 criteria reached consensus with a score of 7 or higher by more than 80% of the participants. The remaining eight proposed criteria did not reach consensus, and six were subsequently dropped after discussions among the core group members. The remaining two criteria were revised based on feedback and suggestions from participants. See Table 2. These two statements were sent to 63 individuals. The response rate for the second round was 65%, with 41 participants providing feedback. However, the two revised statements did not meet the criteria for consensus. As a result, both statements were removed from consideration, resulting in a set of 14 criteria.

**Table 2:** Final Criteria Developed for Hospital Palliative Care Team Referral in a Cancer Setting

Domain	Criteria No.	Recommended Criteria
Cancer-related	**1**	Stage 4 cancer
	**2**	Prognosticated to have life expectancy of < 6 months
Treatment-related	**3**	Failed two or more lines of treatment
Symptom related	**4**	Performance status ECOG ≥ 3
	**5**	Multiple symptoms (including pain) and co-morbidities affecting patients QOL/cancer-directed treatment
	**6**	≥ 4 score on the distress thermometer
Patient and Family request	**7**	Requested palliative care
	**8**	Refused cancer-directed treatment
	**9**	Requested limitation of life-sustaining therapies or hastened death
End-of-life Care	**10**	Perceived need for end-of-life care planning

This list of 14 statements was further reviewed and consolidated to create a simplified and easy-to-understand guide for oncologists. The “stage of disease” and “life expectancy” domains were combined into a single “cancer-related” domain. The “Brain metastases and leptomeningeal disease” criteria have been incorporated into the “stage 4 disease” criterion, and “multiple symptoms/comorbidities affecting further cancer-related treatment options” have been merged with “symptoms-related domain affecting QOL.” The “referral for pain” criterion has also been integrated into symptom-related domains. Performance status and distress criteria were combined with the aforementioned criteria to form a “symptom-related” domain. This process resulted in a list of ten final criteria, organised into five domains, as outlined in Table 2  

**Table 3:** Final Criteria Developed for Hospital Palliative Care Team Referral in a Cancer Setting

Domain	Criteria No.	Recommended Criteria
Cancer-related	1	Stage 4 cancer
	2	Prognosticated to have life expectancy of <6 months
Treatment-related	3	Failed two or more lines of treatment
Symptom related	4	Performance status ECOG ≥3
	5	Multiple symptoms (including pain) and co-morbidities affecting patients QOL/cancer-directed treatment
	6	≥ 4 score on the distress thermometer
Patient request	7	Requested palliative care
	8	Refused cancer-directed treatment
	9	Requested limitation of life-sustaining therapies or hastened death
End-of-life Care	10	Perceived need for end-of-life care planning

## Discussion

Using the modified Delphi survey with oncologists as stakeholders, we developed a list of ten implementable criteria for referral to a hospital palliative care team in a cancer setting. Over 90% of oncologists supported the criteria that focused on managing physical distress, such as pain or other symptoms that impact quality of life and disease-directed treatment (Criterion 5), as well as those with a distress thermometer score of 4 or higher (Criterion 6), given that symptom management lies at the core of palliative medicine. Additionally, oncologists recognised the importance of specialist palliative medicine in end-of-life care planning and provision (Criterion 10).

The relevance of the cancer stage as a criterion was unanimously agreed upon; however, there was some discrepancy regarding the cut-off for referral. While there was a strong consensus for stage 4 cancer (Criteria 1), this was not the case for locally advanced cancer (stage 3 and above). This discrepancy can be attributed to targeted therapies for stage 3 cancers, such as the cervix, breast, and lung, which have significantly improved patient survival rates [17–19]. For example, research has shown that administering Durvalumab after chemoradiotherapy in stage 3 non-small cell lung cancer results in a significant improvement in progression-free survival for patients. In contrast, similar research on stage 4 cancer did not reach statistical significance [20].

In the context of referral criteria, treatment failure was identified as a factor, but the threshold was set at two or more lines rather than one (as per Criteria 3). With advances in chemotherapy, immunotherapy, and targeted therapies, oncologists now have multiple lines of disease-directed treatments [21]. While chemotherapy continues to be a cornerstone of cancer treatment, the symptom burden linked to cancer therapies underscores the importance of early palliative care referral to enhance quality of life and inform decision-making [22]. It is essential to note that our setting is not representative of the entire Indian population, as the study settings catered to patients from mid to high socioeconomic classes in the private sector. Despite this, oncologists recognised the importance of early integration of palliative care, particularly concerning physical distress and end-of-life care, as evidenced by the lack of consensus for stage 3 cancers and patients failing first-line chemotherapy.

When a patient or their family expresses a desire to limit life-sustaining treatment or hasten death (as defined by Criteria 9), it may also indicate the presence of an underlying physical, psychological, social, or spiritual distress [23]. In such instances, involving a palliative medicine specialist who can provide adequate symptom relief, offer comfort to the family, and help them set realistic expectations is essential [24]. Depending on the circumstances, a psychologist or spiritual advisor may also be involved [25]. Given their demanding schedules, oncologists may not always have enough time to engage in these complex interactions and counselling sessions [26].

Although hospital and ICU admissions, as well as emergency or unplanned admissions, can be seen as indirect indicators of increasing symptom burden and poor tolerance of therapy, they are not widely recognised as triggers for referral to palliative medicine by most oncologists, despite being included in NCCN criteria [27]. In the modern landscape of oncology, where patients undergo multiple lines of chemotherapy and immunotherapy-related toxicities, emergency visits can be highly unpredictable [28, 29]. This may explain why our oncologists might not have given much consideration to this criterion for palliative care referral. However, research has demonstrated that palliative care interventions, such as advance care directives and community-based palliative care for home care, can reduce emergency and ICU visits, length of stay in ICU, and the likelihood of dying in the hospital [30, 31].

There was one area where agreement could not be reached: the involvement of palliative care in cases where a patient with cancer is not informed of their diagnosis or prognosis by their family or caregivers, who believe they are protecting the patient from adverse information. In situations where collusion hampers discussions about treatment plans or end-of-life care, involving palliative care specialists might be a viable option [32]. Studies have examined strategies used by palliative care physicians to reduce collusion in patients with advanced-stage cancer [33]. However, over 45% of oncologists in our setting believed that specialist palliative care played a minimal role in such cases. Many thought they had a longer relationship with the patient and family and did not see the need to refer them to palliative care specialists to break the collusion. Moreover, it might not always be appropriate to refer patients who are unaware of their underlying condition to specialist palliative care [34].

### Strengths and limitations

The Delphi technique is a valuable tool for reaching consensus in decision-making related to national and institutional guidelines, especially when international standards cannot be followed due to differences in healthcare infrastructure [35]. In our study, we employed a modified version of the Delphi method to gather insights into oncologists’ perspectives on the appropriate timing for referring patients to specialist palliative care in a private tertiary care setting. Our research involved a diverse group of oncologists, including those in medical, surgical, radiation, paediatric, and haematology-oncology specialities, who are responsible for admitting and referring patients with cancer across various care areas, such as outpatient, inpatient, emergency units, and critical care units. These professionals serve as crucial gatekeepers, making decisions to ensure the best possible outcomes for their patients.

The study’s participants were clinicians working in private-sector hospitals, so the findings might not be fully applicable to the public sector. Additionally, due to varying health infrastructures and differing levels of community readiness and acceptance of palliative care, the results may be more relevant for developing nations than for developed countries. It is essential to note that the study only included doctors at the consultant level and above, as decision-making and referrals typically occur at this level. Future research could involve similar exercises or structured group discussions with fellows, nurses, and other stakeholders to assess the acceptance of these guidelines.

### Clinical practice implications

This study, conducted by a group of private tertiary care hospitals in India, aimed to establish a standard care pathway for patients with cancer who require specialist palliative care. Typically, clinician decision-making is the primary criterion for referrals to specialist palliative care in most hospitals, leading to the underutilisation of palliative medicine services. To address this, the study suggests using checklists to improve health-related quality outcomes and raise awareness among oncologists about the importance of providing specialised palliative care. However, creating these criteria is just the initial step. The real challenge lies in implementation and overcoming attitudinal and logistical barriers through continuous efforts from administrators and the palliative care team. Further studies may be necessary to identify the most effective referral criteria that can enhance quality of life, decrease healthcare utilisation, and be appropriately adopted by clinicians across outpatient, inpatient, critical care, and emergency settings.

### Impact on our practice

The department disseminated these documents among cancer teams, which have since become an essential part of institutional guidelines and standard operating procedures for best oncology practices. To promote the systematic implementation of these guidelines, we launched a quality improvement project that incorporates palliative care into oncology practice. It involved disseminating the established criteria and encouraging compliance among the cancer teams.

## Conclusion

We standardised the criteria for referring patients with cancer to HPCT through a review of the literature, and a consensus was reached using a modified Delphi process. A consolidated set of ten referral criteria across five domains: Cancer-related, Treatment-related, Symptom-related, Patient and Family Preferences, and End-of-Life Care, was developed. These criteria can serve as a preliminary framework to improve the integration of palliative medicine into cancer care. Additional empirical testing is needed to strengthen its clinical relevance and effectiveness.

## Data Availability

Most data generated or analysed during this study are included in this published article. The datasets generated and analysed in this study are stored securely in the data system of the principal investigator and available from the corresponding author on reasonable request.
